# A comparison of various MRI feature types for characterizing whole brain anatomical differences using linear pattern recognition methods

**DOI:** 10.1016/j.neuroimage.2018.05.065

**Published:** 2018-09

**Authors:** Gemma C. Monté-Rubio, Carles Falcón, Edith Pomarol-Clotet, John Ashburner

**Affiliations:** a FIDMAG Germanes Hospitalàries Research Foundation, Avda. Jordà 8, 08035, Barcelona, Spain; b Fundació ACE. Institut Català de Neurociències Aplicades, Marqués de Sentmenat 57, 08029, Barcelona, Spain; c Barcelonaβeta Brain Research Center, Pasqual Maragall Foundation. Barcelona, Carrer de Wellington 30, 08005, Barcelona, Spain; d CIBER en Bioingenieria, Biomateriales y Nanomedicina (CIBER-BBN), Spain; e Wellcome Centre for Human Neuroimaging, UCL Institute of Neurology, 12 Queen Square, London, WC1N 3BG, UK

**Keywords:** Pattern recognition, Gaussian process, Diffeomorphism, Model selection, Scalar momentum, Pattern recognition, Structural MRI, VBM

## Abstract

There is a widespread interest in applying pattern recognition methods to anatomical neuroimaging data, but so far, there has been relatively little investigation into how best to derive image features in order to make the most accurate predictions. In this work, a Gaussian Process machine learning approach was used for predicting age, gender and body mass index (BMI) of subjects in the IXI dataset, as well as age, gender and diagnostic status using the ABIDE and COBRE datasets. MRI data were segmented and aligned using SPM12, and a variety of feature representations were derived from this preprocessing. We compared classification and regression accuracy using the different sorts of features, and with various degrees of spatial smoothing. Results suggested that feature sets that did not ignore the implicit background tissue class, tended to result in better overall performance, whereas some of the most commonly used feature sets performed relatively poorly.

## Highlights

• We tested various types of features, derived from segmented T1-weighted MRI, according to their ability to be used by a linear pattern recognition method to predict age, sex, BMI, and diagnostic status.
• Scalar momenta, and other methods that do not ignore the implicit background class provided more effective strategies.
• The most widely used feature type, namely “modulated” GM, was found to be relatively ineffective.

## Introduction

A common goal of neuroimaging research involves identifying morphometric alterations associated with particular diseases. For example, thousands of studies have involved applying the Voxel-Based Morphometry (VBM) technique (Wright et al., 1995; Ashburner and Friston 2000, 2001) for comparing brain anatomies. With VBM, the aim is to test a hypothesis at each voxel using multiple linear regression (“mass-univariate statistics”). Multiple linear regression is a special case of the general linear model, which is a framework that also encompasses multivariate approaches (such as MANOVA and MANCOVA) that deal with multiple independent and dependent variables. With images, there are thousands or even millions of dependent variables,^1^ so many recent developments have been based on pattern recognition and other machine learning approaches that provide principled ways of dealing with the “curse of dimensionality”.

Methods that treat images as properly multivariate may be better able to obtain characterizations of differences among populations that are accurate enough to actually separate individuals into their respective populations. This greater accuracy may lead to more rapid translation from basic research into clinical applications (Ashburner and Klöppel, 2011). Such applications yield interesting predictions based on more accurate characterizations of differences between populations of subjects (Schrouff et al., 2013; Sabuncu and Konukoglu, 2014). In the last ten years, pattern recognition techniques have been widely applied to structural data, mainly for predicting clinical status at the individual level (Klöppel et al. 2008, 2012; Costafreda et al., 2009; Nieuwenhuis et al., 2012; Mourao-Miranda et al., 2012). A number of authors have suggested that pattern recognition could aid in clinical decision-making and treatment development (Feinstein et al., 2004; Frisoni et al., 2010; Ho et al., 2003).

These methods aim to capture the entire patterns of difference that best separate subjects into groups, or predict some continuous variable of interest. Pattern recognition approaches attempt to learn a relationship between feature data (e.g., preprocessed MRI scans) and sets of corresponding labels (e.g., ages, or disease status). After learning such a relationship, the same mapping should be able to predict the label for new cases, given the image features.

Problems are typically treated as regression or classification, depending on whether the output is continuous or discrete, respectively. There are many different algorithms for pattern recognition, but some of them (e.g. convolutional networks) would only be appropriate for making whole-brain predictions from extremely large sets of training data. Kernel methods, such as Support-vector Machines, have been widely used for the sorts of tasks described in this paper. The high-dimensionality of the feature sets, compared to the usually low numbers of images, means that linear approaches generally perform at a similar level to nonlinear approaches, while retaining much more interpretability (Chu et al., 2011). In the current work, a linear formulation of the Gaussian processes (GP) approach for classification and regression (Rasmussen and Williams, 2006) was used, which is a kernel-based approach set in a Bayesian framework. GP was initially developed for regression (Williams and Rasmussen, 1996), and can be conceptualized as a Bayesian extension of linear regression (Bishop, 2006). They achieve similar performance to Support Vector Machines (SVM) for neuroimaging data (Schrouff et al., 2013) with the advantage that they make probabilistic predictions. It is also possible to obtain a marginal likelihood measure, which can be used for comparing models without resorting to cross-validation.

There are many different ways to represent input features derived from brain MR scans. The most widely used approaches have involved pre-processing structural MRI scans in the same way as for a conventional VBM analysis (Wright et al., 1995; Ashburner and Friston, 2000) but then applying a pattern recognition technique. One aim of this paper is to assess whether or not this is a good approach to use. Kernel methods, such as GPs, require some measure of similarity between anatomies. There are many alternative ways of encoding this similarity, and the overall goal of this paper is to assess what types of approach are most effective.

Generally, the best way to increase the accuracy of pattern recognition methods is to use more training data, but data alone is not enough, no matter how much of it is available (Domingos, 2012). The use of suboptimal features limits the accuracy with which predictions may be made and wastes valuable training data. It is an appreciation of the No Free Lunch Theorem (Wolpert, 2002) that allows us, when confronting practical pattern recognition problems, to focus on the aspects that matter most – prior information, data distribution, amount of training data and cost or reward functions. This scenario leads to a strong motivation for exploring different types of features, how they encode information and how well they generalize to other tasks. No particular feature representation is expected to be best for everything, and one would expect those features most closely associated with the underlying biological process to best encode the important differences between populations. However, there may be general feature engineering principles that tend towards reducing the amount of injected noise.

Some previous studies have contributed towards compiling benchmark results. Sabuncu and Konukoglu (2014) applied three classes of pattern recognition algorithms to commonly used types of structural features derived from publicly available datasets of MR scans, to predict an array of clinically relevant variables. Their results suggested that the biological footprint has a strong influence on prediction performance and that the choice of features can impact the results more than the pattern recognition algorithm itself. Cuingnet et al. (2011) conducted replications of ten classification strategies from the literature, using publicly available scans of subjects with and without Alzheimer's disease. One of the conclusions of this work was that processing structural data using Dartel (Ashburner, 2007), implemented in SPM5, could sometimes improve classification. Other studies have also focused on how well the deformations can be used to distinguish between populations (Lao et al., 2004). Special attention is often paid to predicting age. For instance, Franke et al. (2010, 2012) predicted ages, also using results from their VBM8 toolbox^2^ for SPM8. The work in Franke et al. (2010) shares commonalities with the current one, as they both used T1-weighted images from healthy subjects to predict their ages using a kernel method.

We consider that the description of differences among populations of subjects that is closest to the truth is the one that leads to the most accurate predictions of class membership. This work is an exploratory analysis of several strategies to determine general principles concerning the types of image feature representations that are most effective for whole-brain kernel-based methods, and in situations where the expected differences are distributed throughout the brain. This involves applying GP machine learning approaches, using a number of sets of features, derived from the same subjects' scans, in order to predict a number of different target variables. The hope is that this should allow an effective feature representation to be selected, prior to further work using data from different populations of subjects. When machine learning is applied to relatively small, but valuable, datasets from patient populations, it is important to determine how best to do this beforehand. It would be very poor science to try lots of methods, and selectively report only those that worked the best.

While this work is intended as an exploratory analysis, there may be good theoretical reasons (stated in Ashburner and Klöppel, 2011) for a feature type known as “scalar momentum” being particularly effective. Jacobian-scaled grey matter is one of the more widely used feature sets for pattern recognition, but evidence is accumulating that suggests that it may not be especially effective. The study by Franke et al. (2010) reported that more accurate inter-subject registration did not necessarily lead to greater predictive accuracy when using Jacobian-scaled grey matter. Also, a comparison of Radua et al. (2014) suggested that VBM was more sensitive when not using Jacobian scaling. These findings appeared counter-intuitive and provided some of the motivation for this work. In addition to scalar momentum and Jacobian-scaled grey matter, a few additional related feature types are also included in this work in order to better understand the differences in behavior between the two main features of interest.

## Methods

Relatively large public datasets with respective demographic variables were used in this work. Several sets of features were derived from the image data, using VBM-type pre-processing. With them, different analyses were carried out to explore the performance dependency on the features: GP regression (implemented as a Bayesian ridge regression analysis) was used to predict age and body mass index (BMI), and GP classification was used to predict gender and diagnostic status. Support-vector machines were also applied to the classification tests to assess whether the GP results generalize to at least one other linear classification method.

### Datasets

Comparisons of different feature types were made using three different datasets, the demographics of which are summarized in Table 1. Initial work only involved the IXI dataset, but reviewers requested further comparisons to be made, so the COBRE and ABIDE analyses were also included.

i IXI Dataset

**Table 1:** Demographics of the samples used after datasets were examined.

dataset	simple size	age (years)	gender (male/female)	height (cm)	weight (kg)
**IXI**	562	46.85 ± 16.40	249/313	169.38 ± 9.61	71.19 ± 13.77
**ABIDE**	1102	17.08 ± 8.06	163/939	–	–
*controls*	571	17.10 ± 7.72	99/472	–	–
*ASD patients*	531	17.06 ± 8.41	64/467	–	–
**COBRE**	146	36.97 ± 12.78	37/109	–	–
*controls*	74	35.82 ± 11.57	23/51	–	–
*Sch. patients*	72	38.16 ± 13.80	14/58	–	–

The **IXI** data set^3^ consists of a variety of MR images from nearly 600 normal, healthy subjects with their respective demographic information. Only the T1-weighted images were used. MRI data were acquired in three different scanners, two of which were 1.5 T and one was 3 T. Age, gender and BMI were used as targets to predict. Some “data scrubbing” was performed by identifying variables that seemed less likely to be accurate. In all datasets, weights listed as below 40 kg and over 110 kg, or heights of below 150 cm and over 200 cm were excluded from the analyses.

ii COBRE Dataset

The Center for Biomedical Research Excellence^4^ (COBRE**)**, provides structural and functional MR data from 72 patients with Schizophrenia and 75 healthy controls. For this study, only structural MRI data were used. All subjects were screened and fulfilled the criteria for inclusion, which are detailed in the website, as well as the acquisition parameters. Regarding this dataset, age, gender and diagnostic status were used as targets to test the concordance between efficiencies from the feature types at each independent dataset.

iii ABIDE I Dataset

The Autism Brain Imaging Data Exchange^5^ (ABIDE) initiative aggregates structural and functional MR data collected from laboratories around the world to contribute to the study of autism. MRI data from the first ABIDE initiative, ABIDE I, was used for the current work. ABIDE I data comes from 17 international sites, involving 539 individuals with Autism Spectrum Disorder (ASD) and 573 typical controls. Shared data consist of resting state fMRI, and anatomical MRI, but only MRI data was used for this work (see the corresponding website for further details). In this case, gender and diagnostic status were used as the target to predict. The ABIDE dataset was very variable, with some scans having missing cerebella. Because of this, a strategy to automatically exclude the 20 greatest outliers was adopted. These outliers were identified from the rows (or columns) of the weighted sum of all the dot-product matrices (see later).

### Preprocessing

Each of the three datasets was preprocessed independently. The T1-weighted images were visually inspected for possible artifacts, and approximately aligned (translations) with the SPM template data. Next, a VBM-type pre-processing was conducted. The segmentation algorithm of SPM12 (with default settings) was used for segmenting GM, WM and CSF tissue types from the native images. It is based on the algorithm presented in (Ashburner and Friston, 2005), but makes use of additional tissue classes, and incorporates a more flexible image registration (see appendix of Malone et al. (2015)). Following tissue segmentation, inter-subject registration of GM and WM tissue types was performed using the SPM12 Geodesic Shooting Toolbox (Ashburner and Friston, 2011). This is a tool for modeling shapes of the brain, based on the diffeomorphic registration framework (M. Miller et al., 1997; Grenander and Miller, 1998; M. I. Miller, 2004). A variety of types of features were derived from the registered data and the encoded deformations.

Previous work by Klein et al. (2009) compared a number of widely used nonlinear registration algorithms, and found that Dartel (Ashburner, 2007) was one of the more accurate registration tools. That paper did not assess the Geodesic Shooting toolbox (Ashburner and Friston, 2011), which was released later. More recent evaluations (Ashburner and Friston, 2011), using some of the same data as those of the Klein et al. paper (Klein et al., 2009), have shown that Geodesic Shooting slightly outperforms Dartel (and that both can outperform all the other approaches in the paper by Klein et al.).

### Data for structural feature representation

**Fig. 1:**
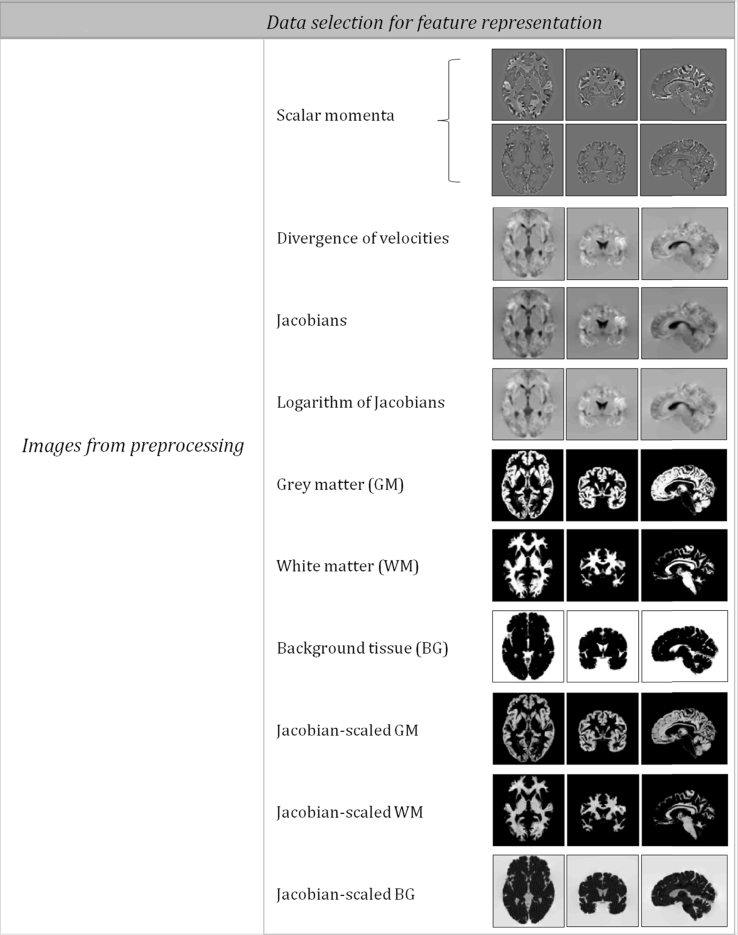
Resulting images from the pre-processing and those obtained from operations, such as the logarithm of the Jacobians and the Background images, BG = 1-(GM + WM). All these images were tested as feature sets for pattern recognition.

Preprocessing outputs were used as features. Each feature representation encodes a different kind of information about the original image data. The field of view of the feature data covers the whole brain, and the features that have been used are listed in Fig. 1.

The simplest feature sets were the warped grey (*c*_1_ ∘ *φ*, where *φ* is the deformation estimated by the geodesic shooting toolbox) and white matter (*c*_2_ ∘ *φ*) and a warped map of the background (BG, constructed by (1 − *c*_1_ − *c*_2_) ∘ *φ*). Each of these was considered alone or together with some of the others.

Jacobian scaled (“modulated”) versions of the tissues were also used as features (|***D***φ|*c*_1_ ∘ *φ*,|***D***φ|*c*_2_ ∘ *φ* and |***D***φ|(1 − *c*_1_ − *c*_2_) ∘ *φ*, where |***D***φ| denotes the Jacobian determinants) either individually or in combination with the others.

The other fields considered were the divergence of the initial velocities (∇∙***v***_0_, see the Appendix for an explanation of the velocities), the Jacobian determinants of the deformations (|***D***φ|) and the logarithms of the Jacobian determinants (log|***D***φ|).

Finally, a feature set referred to as “scalar momentum” (Singh et al., 2010) was also used. These features are described more extensively in the appendix, but consist of |***D***φ|(μ_1_ − *c*_1_(*φ*)),|***D***φ|(μ_2_ − *c*_2_(*φ*)) and |***D***φ|(*c*_1_(*φ*) + *c*_2_(*φ*) − μ_1_ − μ_2_), these fields were not considered individually. Essentially, the use of scalar momentum is a form of generative embedding, as scalar momentum is one form in which diffeomorphic deformations can be parameterized. In generative embedding, pattern recognition effectively uses model parameters as features (Brodersen et al., 2011). A recent report by the Royal Society, entitled “*Machine learning: the power and promise of computers that learn by example*”, suggests that machine learning methods could become more data efficient by encoding the many constraints we know about the real world into them. For anatomical images, the types of constraints to consider are that two brain structures do not occupy the same space, and that volumes, lengths, areas, etc., cannot be negative. The diffeomorphic model used to align the images encodes these constraints, so a generative embedding of model parameters may lead to more effective feature sets. Although there are other ways to parameterize the deformations, scalar momentum, in addition to encoding the deformations, also encodes the residual differences between the registered images and template. This allows a unification of VBM types of approaches (that assess residuals after registration, or Jacobian scaled residuals) with those approaches based on analyses of deformations. A version of the latter approach is widely used in other areas of biology, under the name “statistical shape analysis” or “geometric morphometrics”. Scalar momenta used in this work were obtained from the preprocessing using the Geodesic Shooting Toolbox (Ashburner and Friston 2011), and have been used previously in previous work by Marquand et al. (2013).

### Smoothing

Spatial smoothing of varying degrees was applied to the raw feature images to reduce noise and finer grain anatomical variability. The effect of smoothing was explored in order to find the optimal amount to apply for each feature type. The various feature representations were smoothed over a range of full width at half maxima (FWHM), from 0 mm to 20 mm, with increments of 1 mm.

### Gaussian Process classification and regression

Predictions were carried out using Gaussian Process (GP) machine learning algorithms for regression and classification. The GP classification used an implementation of the expectation propagation approach of Rasmussen and Williams (2006), which is available in the SPM12 software. The regression approach used was an in-house implementation of a Bayesian ridge-regression (from Bishop, 2006), which made use of a singular value decomposition of the kernel matrix. This was equivalent to a Gaussian process model using a dot-product covariance function.

The feature datasets were used as inputs, and transformed into linear kernel matrices using the dot-product, to become the corresponding covariance functions. The kernel matrices were obtained by computing **XX**^T^ from each feature dataset, represented as a set of *N* vectors, each with *k* components (number of voxels), resulting an *N×N* matrix. Thus, the *k* dimensions encoded in each image are reduced to *N*. When spatial smoothing is used, this may be conceptualized as constructing a kernel matrix from **XΣX**^T^, where **Σ** is a Toeplitz matrix that encodes the smoothing.

In the current study, kernel matrices were constructed from the 210 feature sets (10 types of feature with 21 different levels of smoothing) described above. Several new kernels were also studied, which were constructed by adding some of the original kernel matrices together. The tissue class kernel matrices were additionally combined in four different ways: GM + WM; GM + WM + BG; Jacobian-scaled GM + Jacobian-scaled WM; and Jacobian-scaled GM + Jacobian-scaled WM + Jacobian-scaled BG. This sum of kernels was done over all levels of smoothing, so 84 new kernel matrices were added to the initial 210. Note that the kernel matrices were simply added together. This is different from the multi-kernel approach (described later), whereby the optimal positive linear combination of kernel matrices is estimated.

The kernel matrices were used as inputs in a pattern recognition algorithm for regression when the labels were continuous (e.g. age), and for classification when these were discrete (e.g. gender). Gaussian process models were used to make the predictions.

### Model comparison by cross-validation

Generalization performance was assessed using a *k*-fold cross-validation (CV) strategy, which allowed most of the sample to be used during the training stage. A 10-fold CV was used for the IXI dataset, with the same subdivision into folds for all kernel matrices. Five-fold CV was used for the larger ABIDE dataset, whereas for the COBRE dataset, five-fold CV was repeated ten times with different random splits into folds.

For regression, the model predicts the expectation of the corresponding target. The root mean squared (RMS) error was computed for each model, which gives a measure of how well the model generalizes, and allows a comparison to be made between feature sets. Mean absolute errors were also computed.

For classification, instead of predicting the expected mean (and variance) of each target variable, a probabilistic label of belonging to one class or another is predicted. The area under the curve (AUC) of the Receiver Operating Characteristic (ROC) curve was calculated. The AUC is a measure of how well the classifier has performed, being a summary of the performance of the classifier across all decision thresholds (i.e. posterior probabilities). When a classifier makes the perfect discrimination the AUC is 1. For a binary classifier guessing at chance-level, it would achieve an AUC of around 0.5. Classification accuracy was also computed, based on thresholding the probabilities at 0.5.

The Gaussian process approach makes probabilistic predictions, which allows test information to be computed. For the case of binary classification, test information was computed in bits, as described by Rasmussen and Williams (2006). Briefly, if the training labels (**t**) and test labels (t^*^) have values of 0 or 1 indicating membership of the second class, and the predictions of the test labels (**p**) give a probabilities of membership of the second class, then this target information is given by:

$$I=\;\frac{1}{N}\sum_{n=1}^{N}\left(t_{n}^{*}\;\log_{2}p_{n}+\left(1-t_{n}^{*}\right)\log_{2}\left(1-p_{n}\right)\right)-\left(\bar{t^{*}}\;\log_{2}\bar{t}+\;\left(1-\bar{t^{*}}\right)\log_{2}\left(1-\bar{t}\right)\right).$$

For example, given balanced classes, and a binary classification accuracy of 70%, the target information would be 0.119 bits. However, when a system can assign accurate probabilities, it will give a higher target information, even if its binary accuracy is still 70%. A similar information theoretic measure of target information was also computed for the regression, except this used units of nats, rather than bits (as it was computed using log_e_, rather than log_2_). The computation was based on probabilities according to Gaussian distributions encoded by the predicted means and variances.

### Bayesian model comparison

In addition to measures obtained by cross-validation, the log-marginal likelihood of the entire dataset was also computed as a measure of generalization performance. This is a measure of the probability of the targets, given the feature set and hyper-parameters. As the GP models using each feature set involved the same number of estimated hyper-parameters, any adjustments to this measure using (for example) the Bayesian information criterion (BIC) or Akaike information criterion (AIC) should be the same for all cases (although further work could improve the handling of uncertainty in hyper-parameter estimation). Therefore, it may be used for Bayesian model comparison. For comparing one model against another, the Bayes Factor (BF) was used (Kass and Raftery, 1995; Jeffreys, 1961). Given a pair of models with the same number of hyper-parameters, the plausibility of the two different models (*M*_*1*_ and *M*_*2*_) of the data (D) may be (approximately) assessed by

$$BF=\frac{P\left(D|M_{2}\right)}{P\left(D|M_{1}\right)}=\text{exp}\left(\ln\;P\left(D|M_{2}\right)-\ln\;P\left(D|M_{1}\right)\right).$$

The BF can be interpreted by means of the scale defined by Kass and Raftery (1995). This scale varies from 1 to >150, and is divided into blocks (strength of evidence: 1 to 3 ∼ barely worth mentioning; 3 to 20 ∼positive; 20 to 150 ∼strong; >150 ∼very strong.

Identifying a useful feature set is a form of model selection, whereby the aim is to maximize the probability of the data (target variables), given the feature set. Model selection using Bayes factors is now a widely accepted approach in the neuroimaging field, although it still appears to be less readily accepted for pattern recognition applications, where cross-validation still appears to be preferred.

### Support-vector classification accuracy by cross-validation

In addition to the Gaussian Process classification, support-vector machine (SVM)^6^ classification was also used with the same kernel matrices (Gunn, 1997). Rather than do lots of additional cross-validations to search for the optimal value for the *C* setting, a hard margin SVM was used because this has previously been shown to work best for whole brain data (Chu et al., 2011). The idea here was to check whether the general trends obtained using Gaussian Processes also generalized to a more widely used pattern recognition method. AUC and classification accuracy were reported using the same CV scheme as for the Gaussian process classifications, allowing them to be compared between the two classifiers. No attempt was made to fit a sigmoid to the SVM output to obtain probabilistic predictions, as Tipping (2001) showed that this approach was not especially effective, and suggested that it could be costly if used for tasks such as medical diagnosis.

### Multi-kernel learning

Following the analysis using single kernel matrices for each feature set, the behavior of multi-kernel methods was also assessed using the IXI and COBRE datasets, as a few investigators prefer to use this type of approach. The GP framework allows training so that the optimal (positive) weighted combination of kernel matrices is selected using automatic relevance determination. A number of kernel combinations were used, and results plotted with varying degrees of spatial smoothing. The kernel combinations were:

1. A weighted combination of two kernel matrices. The first matrix was computed from the divergences of the initial velocities with no smoothing, whereas the second was computed from the scalar momentum, and examined a range of different degrees of smoothing. This approach is closest to that proposed in Ashburner and Klöppel (2011). This kernel combination is a situation where multi-kernel approaches are more useful, as it allows differential weighting of data of different types or with different units.
2. A weighted combination of three kernel matrices. These were computed from the spatially normalized grey matter, white matter and background, without any Jacobian scaling.
3. A weighted combination of three kernel matrices. These were computed from the Jacobian-scaled spatially normalized grey matter, white matter and background.
4. A weighted combination of two kernel matrices. These were computed from the spatially normalized grey matter and white matter, without any Jacobian scaling. This is intended to replicate an approach that some investigators may currently use.
5. A weighted combination of two kernel matrices. These were computed from the Jacobian-scaled spatially normalized grey matter and white matter. This is intended to replicate the most widespread form of multi-kernel approach.

The multi-kernel approach was applied only to the IXI and COBRE datasets, but not to the ABIDE data.

## Results and discussion

**Fig. 2:**
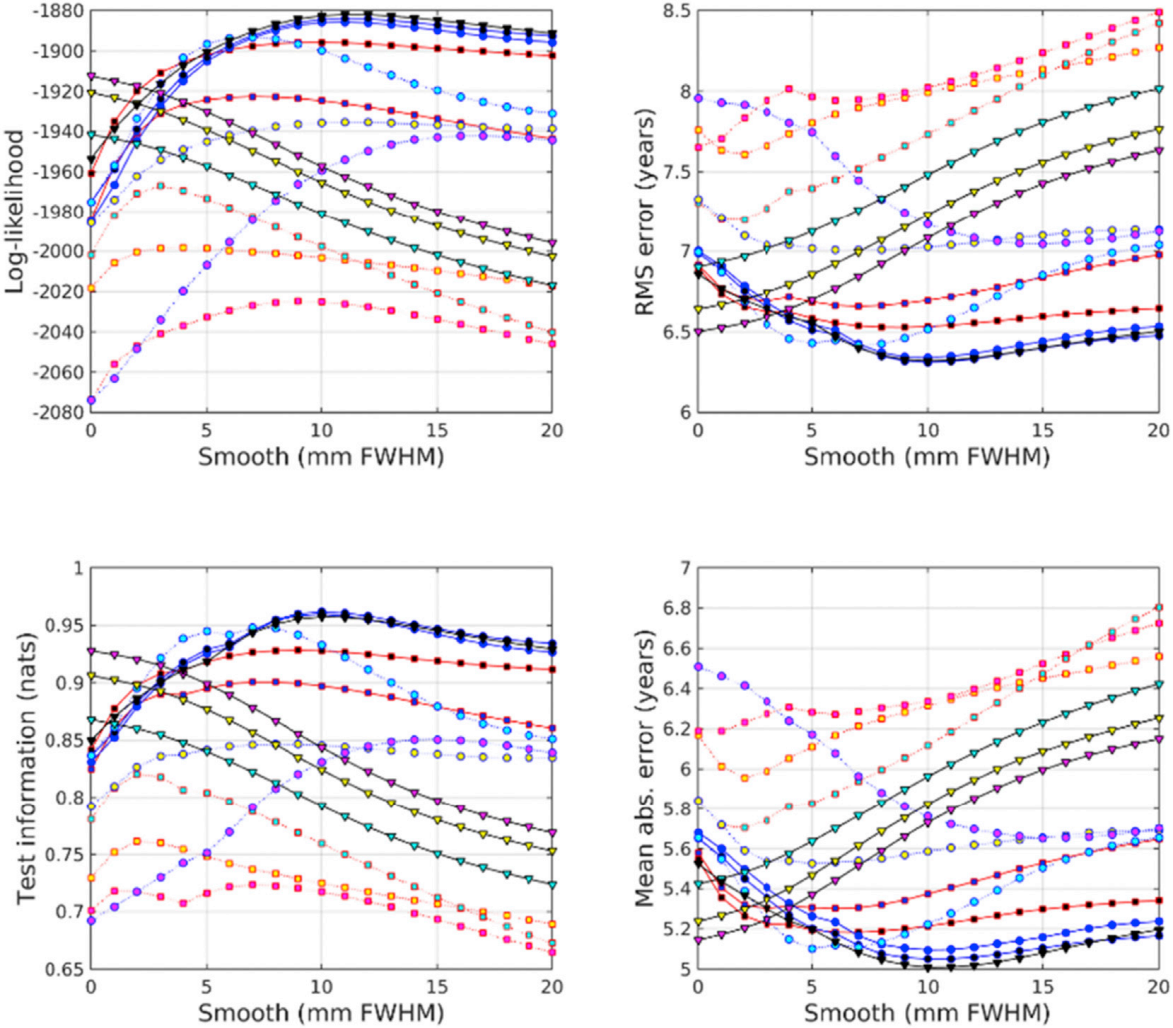
Age predictions from the IXI dataset. See Fig. 10, Fig. 11 for legends.

All accuracies were assessed using test information, which was computed using cross-validation. Results from predicting age from the IXI dataset are shown in Fig. 2. From the feature types used here, age can best be predicted by combining unmodulated GM, WM and BG. This is very closely followed by combining unmodulated GM and WM, and then by scalar momentum. Modulated (Jacobian-scaled) GM, WM or BG by themselves performed poorly. GM, without modulation, gave the best single tissue class performance. Previous work has found that brain ageing follows a specific pattern in which GM volume plays a relevant role with respect the other tissues. The general trend is that GM increases from birth until the age of four and then decreases until the 70s (Pfefferbaum et al., 1994). More recent studies have found that GM decreases linearly with age, while WM did not (Good et al., 2001).

**Fig. 3:**
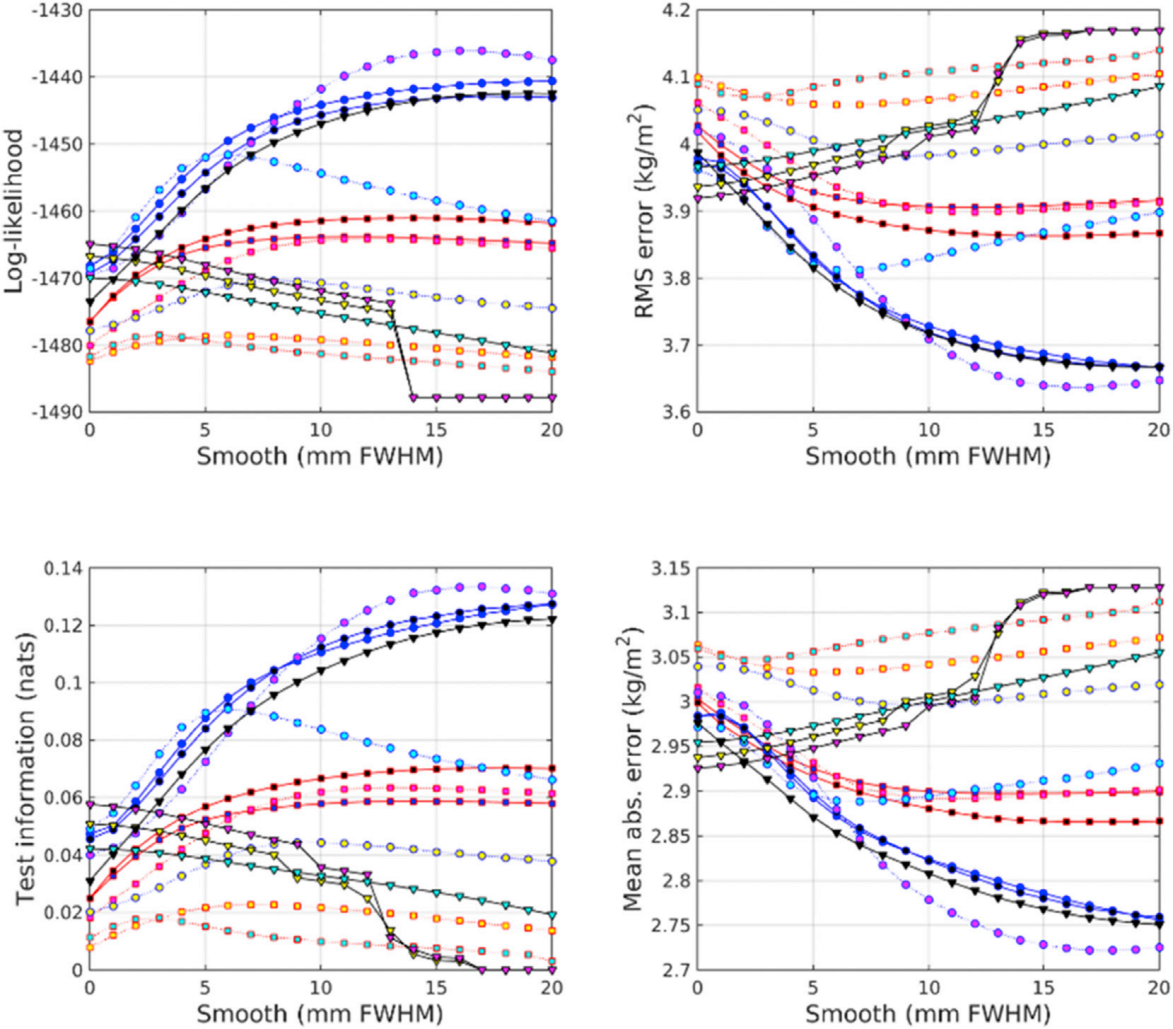
BMI predictions from the IXI dataset. See Fig. 10, Fig. 11 for legends.

Results from predicting BMI from the IXI dataset are shown in Fig. 3. Assessment using test information showed that BMI can best be predicted from unmodulated features. Using WM by itself proved most effective. GM and WM together were second most effective, and GM, WM and BG together were third best. Scalar momentum came fourth. Modulated tissue maps were less effective, although better results are obtained if the BG class is also considered along with the GM and WM. Large amounts of smoothing (around 20 mm) were needed for the best results.

Highest accuracies are generally assumed to be achieved when using features that are more closely associated with the biological process. Previous findings in the literature have indeed established that there is a relationship between BMI and WM (Segura and Jurado Luque, 2009; Seitz et al., 2015; Ho et al., 2003; Ou et al., 2015), although there are discrepancies about whether WM volume is positively or negatively correlated with BMI. In children and adolescents, higher BMI was associated with smaller GM and larger WM volumes without any impact on TIV (Lange, 2012). Karlsson et al. (2013) found that body fat percentage is the critical factor explaining GM and WM volume reductions. Yokum et al., (2012) showed that obese participants had lower total GM and WM volume than lean and overweight participants, but BMI correlated with higher WM volumes in the middle temporal gyrus, fusiform gyrus, parahippocampal gyrus, Rolandic operculum, and dorsal striatum. Haltia et al. (2007) suggested that obesity and dieting are associated with opposite changes in brain structure, and do not exclude the possibility that WM expansion in obesity may play a role in the neuropathogenesis of degenerative brain diseases. Findings suggest a relationship between WM volume and BMI, but the underlying reasons remain unclear.

**Fig. 4:**
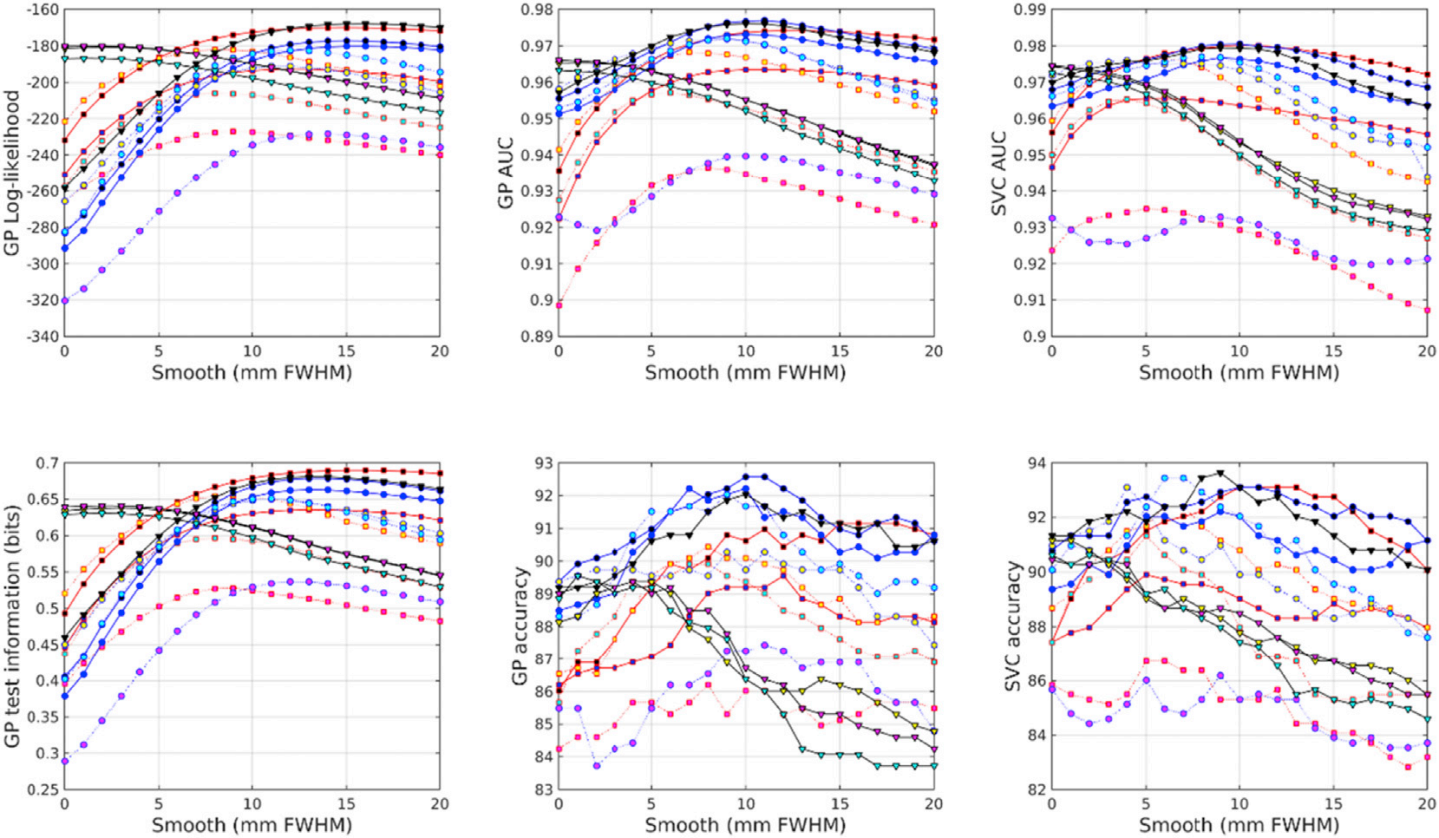
Gender predictions from the IXI dataset. See Fig. 10, Fig. 11 for legends.

Results from predicting gender from the IXI dataset are shown in Fig. 4. The combination of modulated GM, WM and BG proved most effective. Scalar momentum was the second most effective feature set, and the combination of unmodulated GM, WM and BG came third. Features derived from the deformations (Jacobians and divergences) performed reasonably well, which may be because gender can be well predicted by overall head size.

**Fig. 5:**
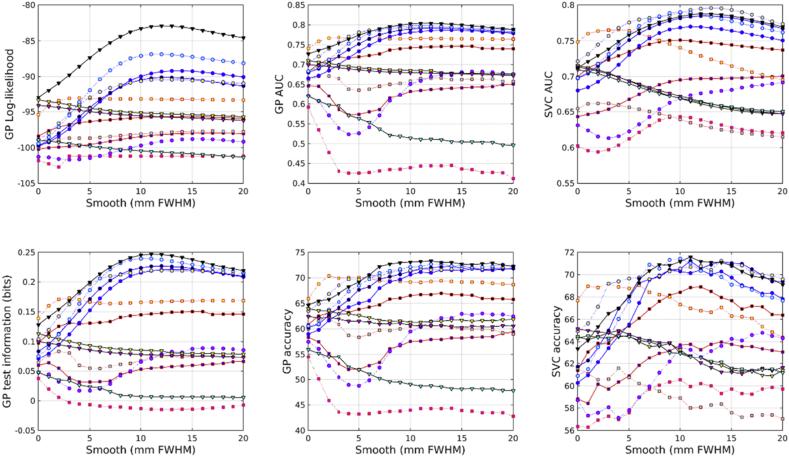
Schizophrenia predictions from the COBRE dataset. See Fig. 10, Fig. 11 for legends.

In the COBRE dataset, the most accurate identification of patients with schizophrenia was achieved using scalar momentum (see Fig. 5). This was closely followed by unmodulated GM, and the combination of GM, WM and BG. Modulated features were generally less effective. For some reason, the marginal likelihoods showed a much greater tendency than test information for favoring scalar momentum. The reasons for this remain unclear, but may be due to the uncertainty with which hyper-parameters are estimated not being a good match for the assumptions underlying BIC or AIC adjustments.

**Fig. 6:**
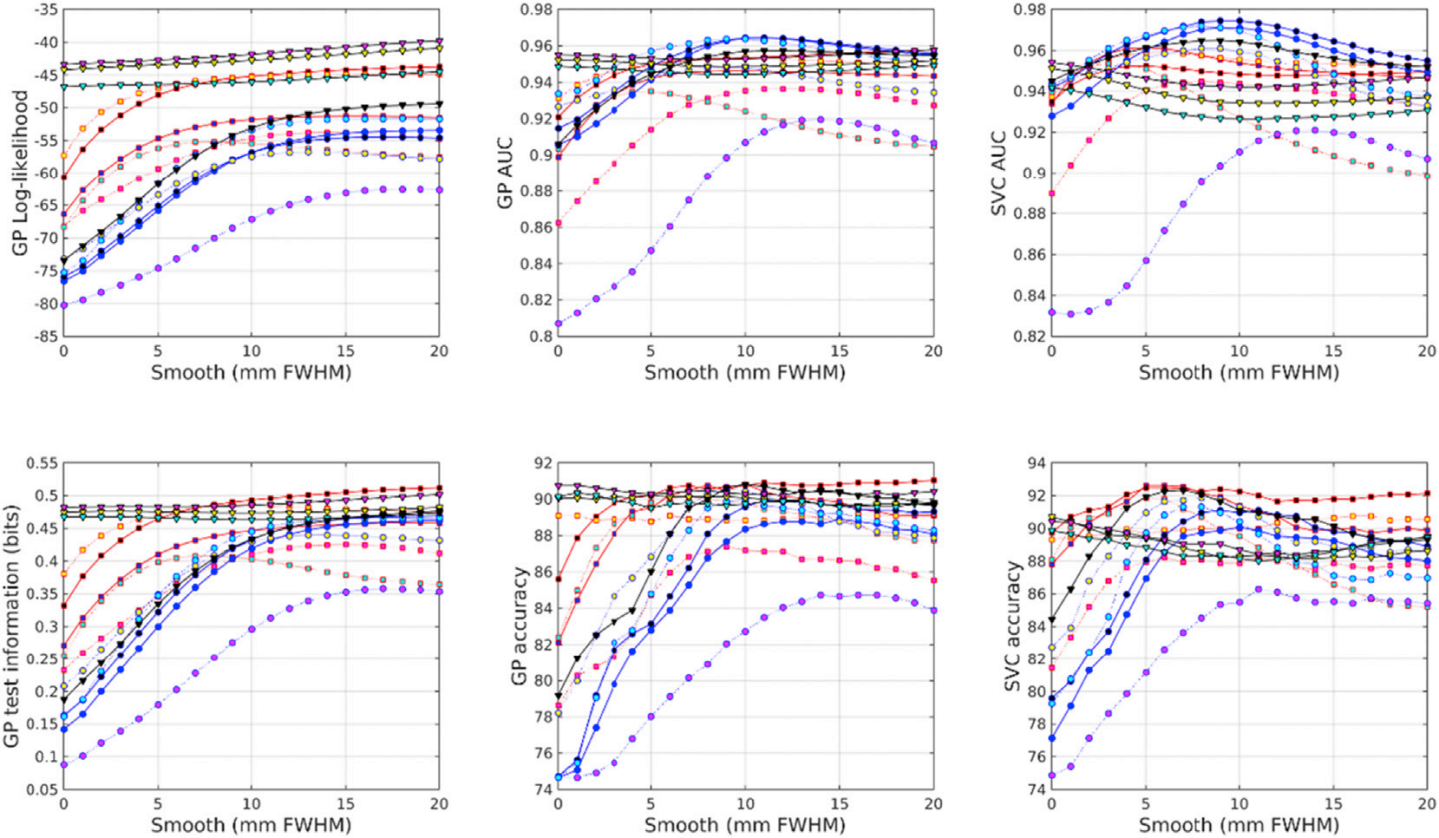
Gender predictions from the COBRE dataset. See Fig. 10, Fig. 11 for legends.

In the COBRE dataset, gender was best predicted by combining modulated GM, WM and BG (see Fig. 6). This was followed by the logarithms of the Jacobian determinants, and then the modulated BG class. Scalar momentum was less effective, coming 5th out of 14. Using a relatively large amount of smoothing seemed to benefit classification accuracy more for this task than for others. For some reason, the overall pattern of accuracies for predicting gender in the COBRE dataset differed from those of the other two. Perhaps the smaller size of the dataset meant that more subtle differences were not recognized and the best features were those that directly encode head size.

**Fig. 7:**
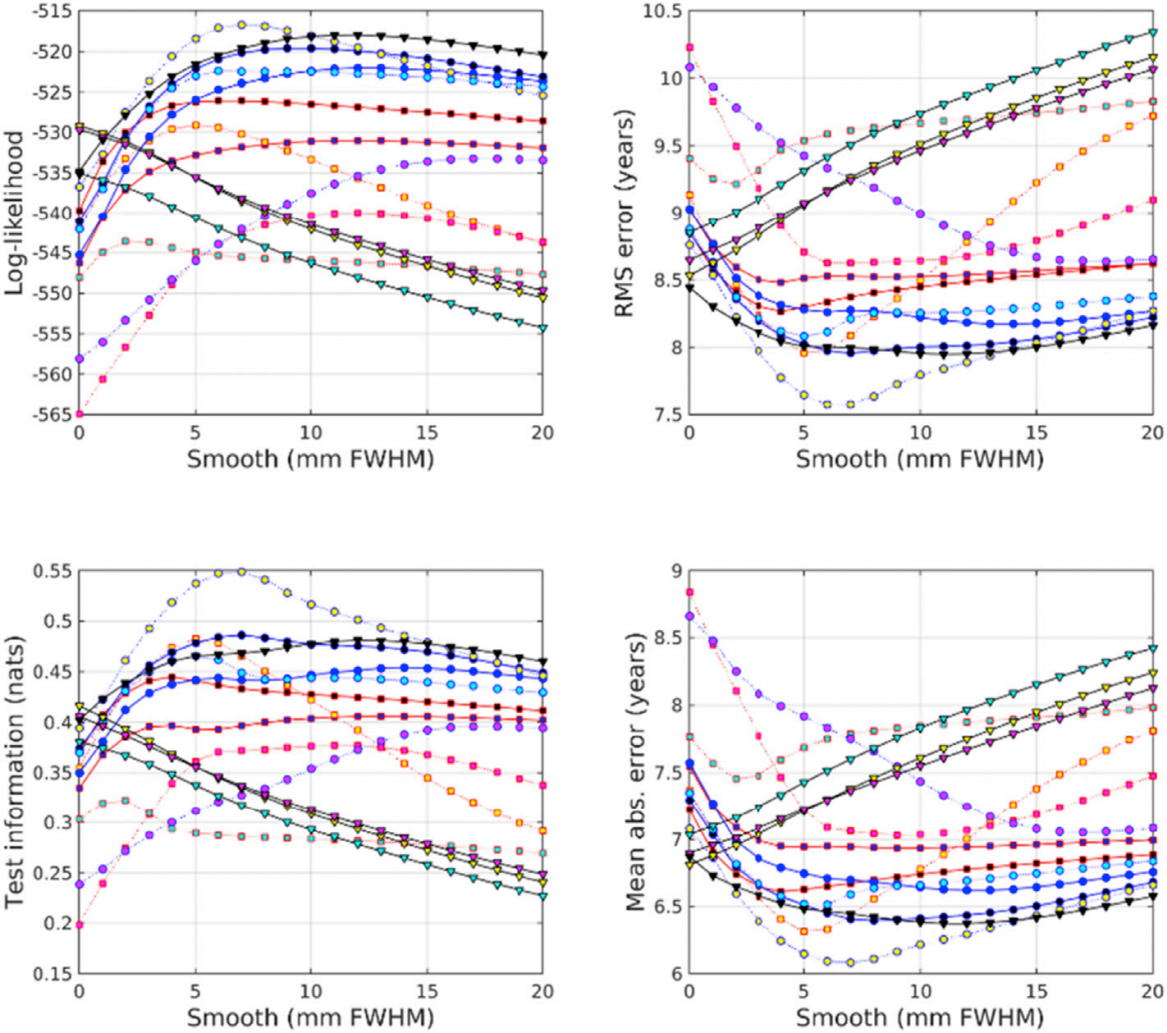
Age predictions from the COBRE dataset. See Fig. 10, Fig. 11 for legends.

The unmodulated BG class seemed to be particularly informative for predicting ages of subjects in the COBRE dataset (see Fig. 7). Unlike the age regression applied to the larger IXI dataset, the best results were obtained from unmodulated BG. The next best performance was from the combination of unmodulated GM, WM and BG. Modulated BG was the third most effective and scalar momentum was fourth.

**Fig. 8:**
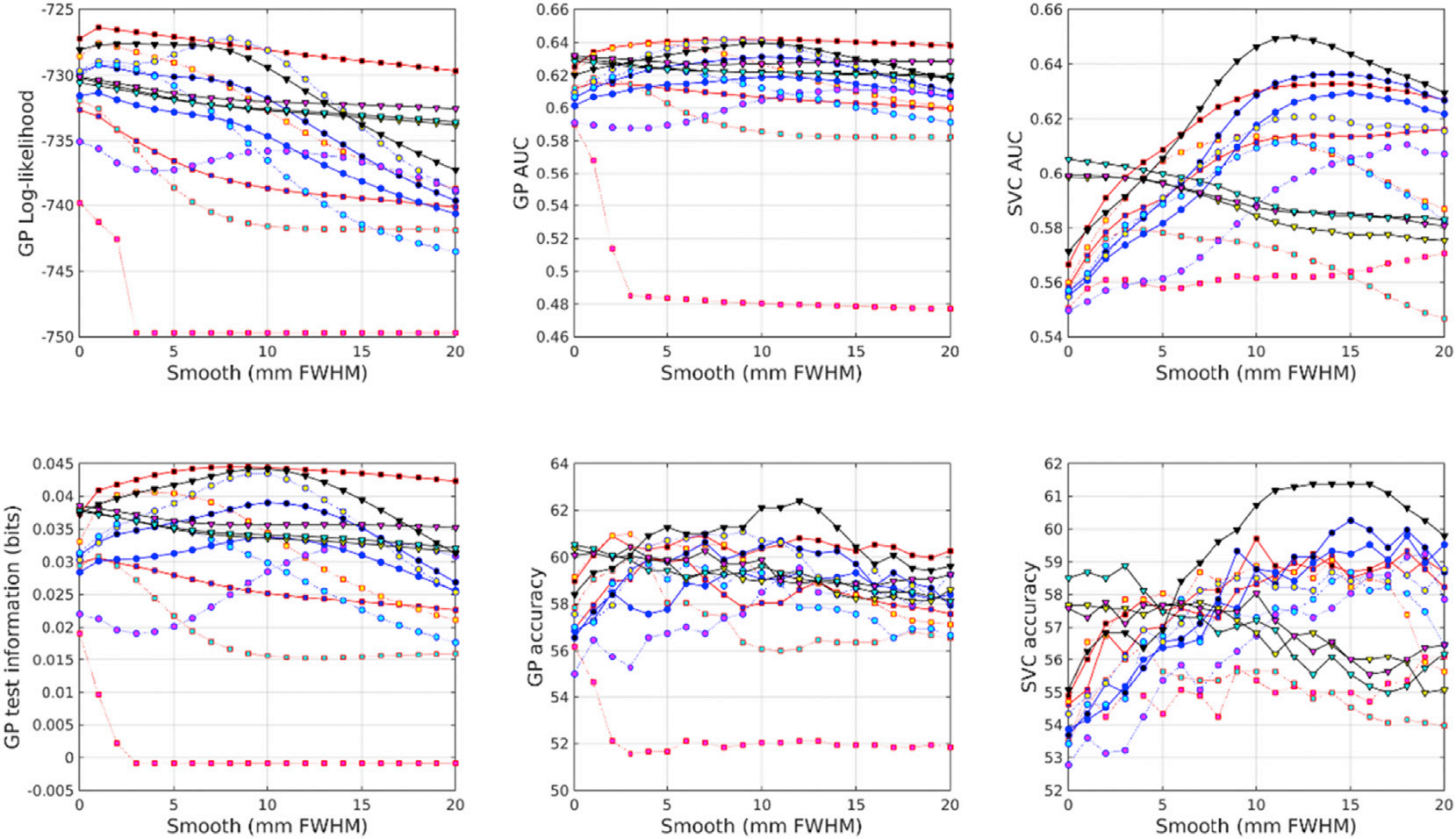
Autism predictions from the ABIDE dataset. See Fig. 10, Fig. 11 for legends.

Identification of individuals with ASD was most accurately achieved using the combination of modulated GM, WM and BG (see Fig. 8). This was followed by scalar momentum and unmodulated BG. By itself, modulated WM as a feature caused the GP classifier to fail completely, whereas SVM handled it rather better. None of the accuracy measures were particularly high. Poor generalizability over the entire ABIDE dataset was previously observed in work by Katuwal et al. (2015), although they achieved good accuracies for within-site classification, which they assumed was due to over-fitting due to using feature selection with small sample sizes.

**Fig. 9:**
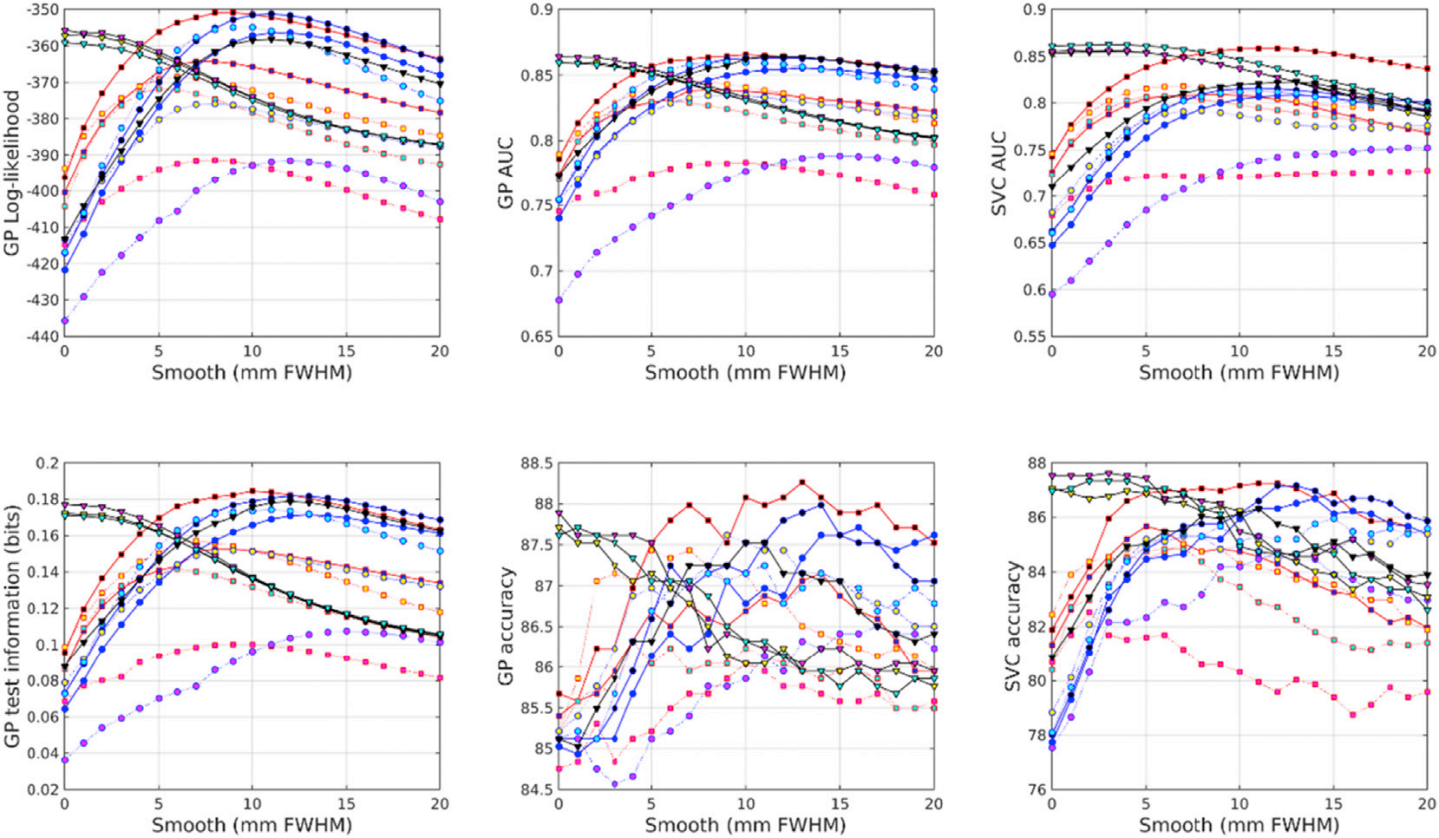
Gender predictions from the ABIDE dataset. See Fig. 10, Fig. 11 for legends.

Gender predictions in the ABIDE dataset followed a similar pattern to those from the IXI dataset, although the overall accuracy was generally lower, probably because of the more variable and lower quality scans than those in IXI. The test information was especially low, which could perhaps be explained by the gender imbalance in the dataset leading to a higher baseline accuracy. The combination of modulated GM, WM and BG performed best. This was followed by combined unmodulated GM, WM and BG, with scalar momentum in third place.

### Summary of results

**Fig. 10:**
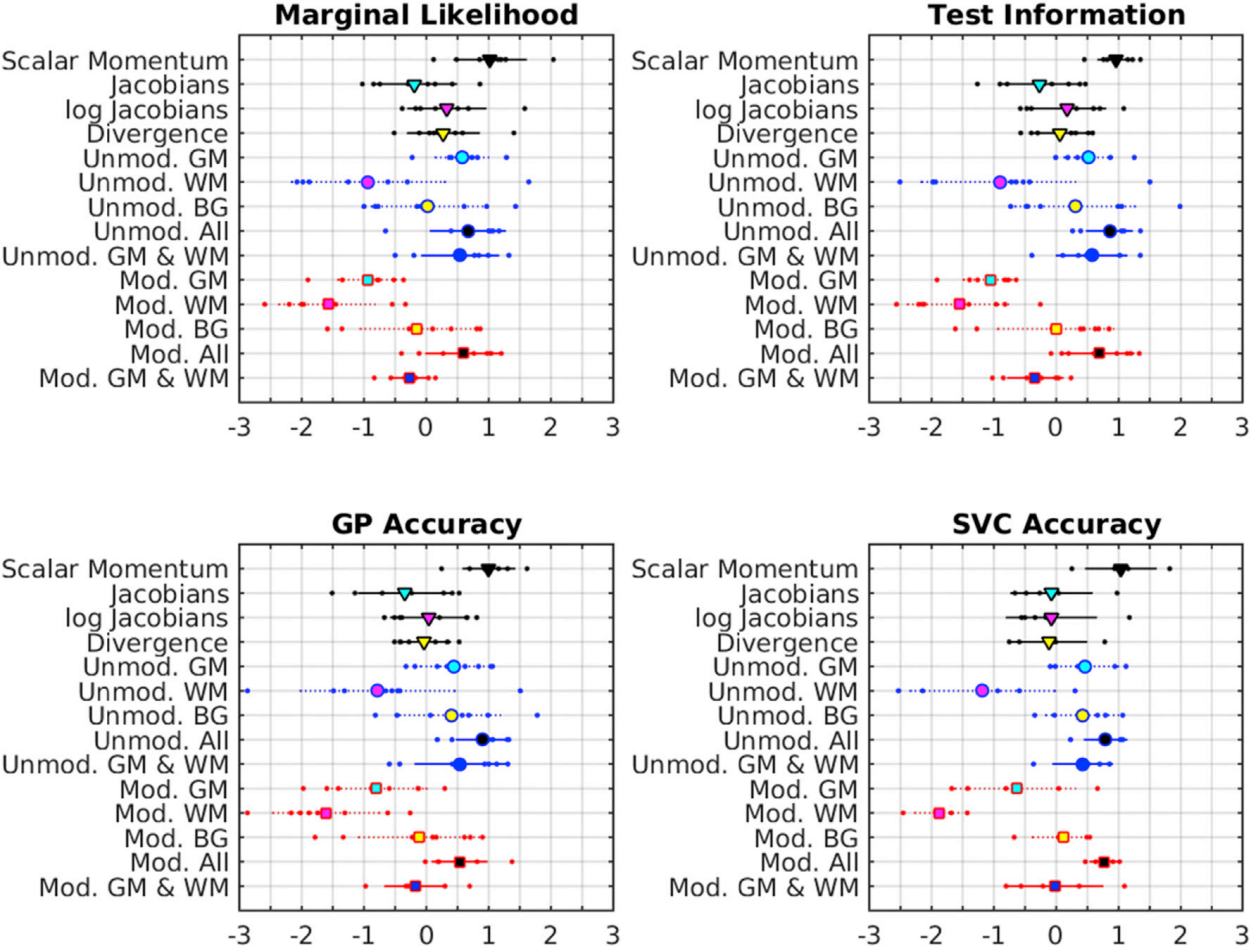
Summary of overall scores. The large markers show the means, whereas the lines indicate ± one standard deviation.

Results are summarized using a number of measures, and are shown in Fig. 10. The marginal likelihood and test information measures were computed for all eight tasks in a straightforward way. For support-vector classification, only accuracy from the five classification tasks was used, whereas the accuracy measure for GP also used the negative of the RMS errors from the regression tasks. For each of the measures, tasks and feature sets, the best predictive performance over the different degrees of smoothing was selected. For each task, the selected measures were standardized by normalizing them to a zero mean Gaussian distribution. This involved subtracting their mean and dividing by their standard deviation. Irrespective of the measure used, scalar momentum performed best on average, although some other feature sets performed similarly.

To assess how well the results generalize over tasks, paired t-tests using the test information measures showed that just using Jacobian-scaled GM as a feature was less effective than scalar momentum (p = 5.2 × 10^−6^, uncorrected for multiple comparisons). Similarly, using Jacobian-scaled GM and WM together, without considering the BG class, was also less effective (p = 7.9 × 10^−4^, uncorrected). However, there was little evidence to suggest that scalar momentum was consistently better than using Jacobian-scaled GM, WM and BG together (p = 0.33). Similarly, the performance of scalar momentum compared to using a combination of unmodulated GM, WM and BG was statistically indistinguishable (p = 0.34), and there was negligible difference between scalar momentum and a combination of unmodulated GM and WM (p = 0.062). Scalar momentum showed fractionally better performance than Jacobians (p = 0.0047, uncorrected), log-Jacobians (p = 0.033, uncorrected) and divergences (p = 0.0064, uncorrected).

There was rough agreement in the effectiveness of the various features, irrespective of whether SVMs or GPs were used. Also, the rankings obtained from using marginal likelihood, test information or simple accuracies followed the same general pattern.

**Fig. 11:**
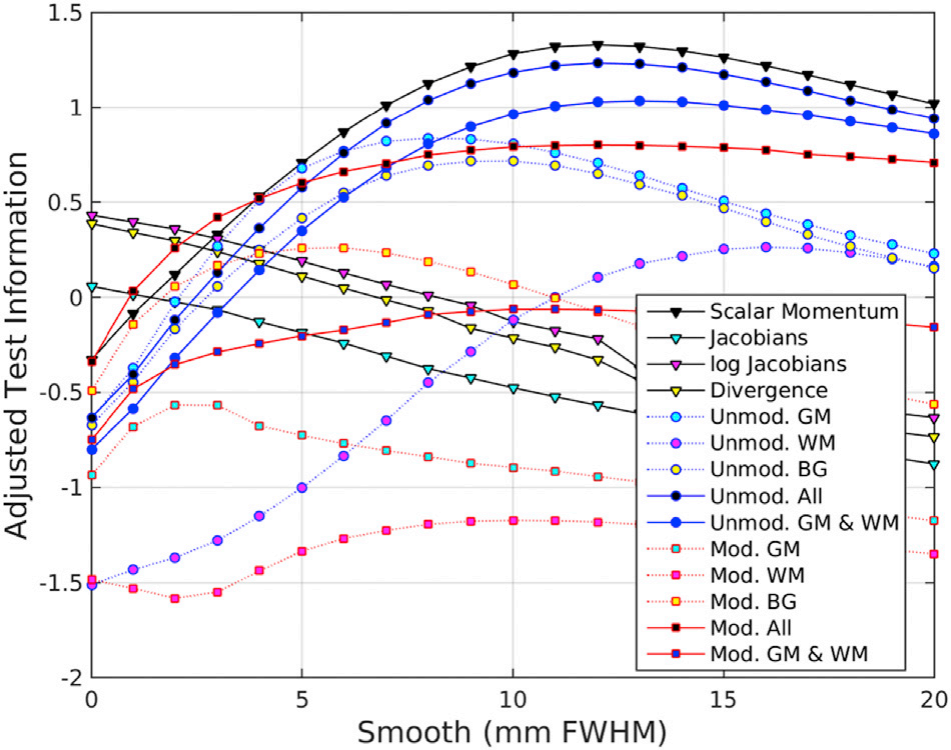
Summary of accuracy measures over different degrees of smoothing.

Another summary of the results is presented in Fig. 11, showing the effectiveness of GPs using features with different degrees of smoothing. This plot was computed by normalizing the test information for each task to a zero mean Gaussian distribution, and computing a weighted average over tasks. Weights for gender classification were 1/3 those used for identifying patients or for BMI regression, whereas those for age regression were scaled by ½. Overall, the best results were obtained using scalar momentum features smoothed by about 12 mm FWHM. Other feature sets using all three tissue classes also performed best using a similar amount of smoothing.

### Multi-kernel learning

**Fig. 12:**
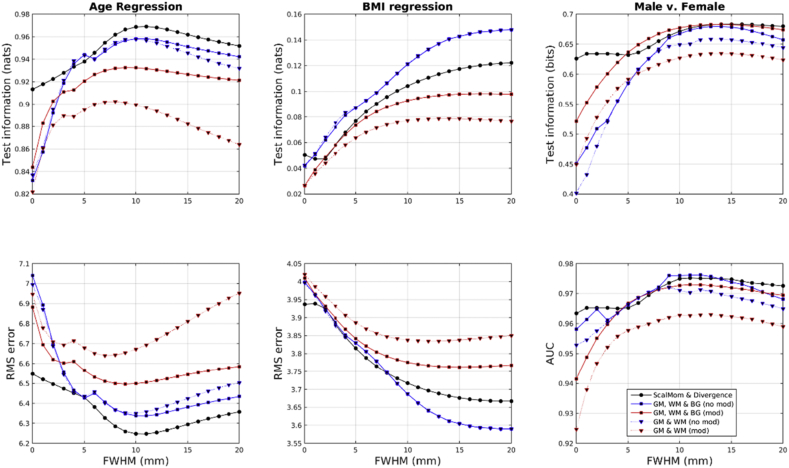
Results from multi-kernel learning applied to the IXI dataset.

**Fig. 13:**
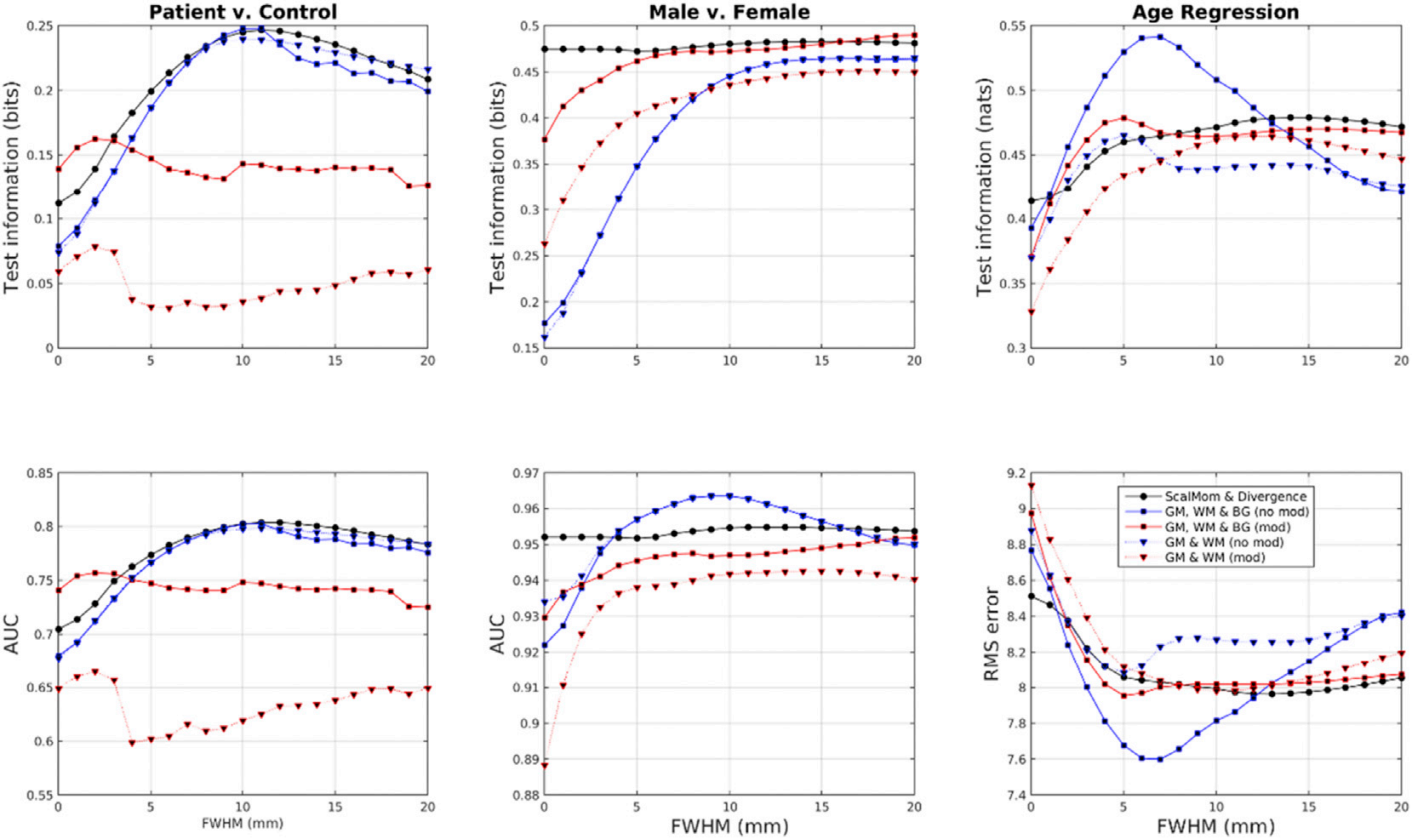
Results from multi-kernel learning applied to the COBRE dataset.

Results from applying multi-kernel learning (MKL) are shown in Fig. 12, Fig. 13. Note that plots of marginal likelihood are not included, as these depend on the number of hyper-parameters in the model. As can be seen, the general trend is that the multi-kernel approaches give results similar to selecting the best of the individual kernel matrices. Sometimes MKL performs slightly better than the best single kernel approach, whereas on other occasions it does not. For the IXI dataset, the first kernel combination (divergence and smoothed scalar momentum, shown as black circles) gave generally reasonable results for the three tasks, although it did not perform as well as the second kernel combination (unmodulated GM, WM and BG, shown as blue squares) for BMI regression.

In contrast to the larger IXI dataset, age regression using the COBRE dataset benefits from being able to give the BG class additional weighting relative to the other tissues. This may be a real effect, although the relatively small differences in log-likelihood for “BG” versus “All”, shown in the plots of Fig. 7, suggest that it could also be down to chance, and the fact that age regression with the IXI data set did not show this pattern adds some support to that idea. The COBRE gender classification task had unbalanced proportions of male and female, which may explain the marked difference between the AUC and the test information results.

Overall, the MKL approach combining scalar momentum with divergences performed reasonably well for all tasks, whereas using just a combination of modulated GM and WM tended to give the worst performance. The combination of divergences and scalar momenta always outperformed the combination of Jacobian-scaled GM and WM. Accuracy measures (not shown) followed a similar pattern to the test information, although the plots were noisier. Note that for the Jacobian-scaled data, the results were generally better when the BG class was also considered (red squares always show better performance than red triangles).

Multi-kernel approaches impose a type of hierarchical structure on the feature sets, such that some features may be weighted more heavily than others. It typically makes much more sense to use a multi-kernel approach when combining features of different types, which may have different units of measurement. For example, it is useful to determine the optimal weighting of different types of data when combining signal from PET and MRI, or when incorporating genetic or demographic information in addition to images. In general though, if there is no *a priori* motivation for assuming a known hierarchical structure, then there is less justification for formulating the problem with a multi-kernel approach. Similarly, sparsity inducing approaches (e.g., those that regularize using LASSO) may be effective for situations where informative signal is scattered over a few isolated voxels. This assumption may hold for some fMRI experiments, but it has very little biological plausibility for the types of anatomical differences this work looked into.

## Conclusions

GP machine learning approaches have been used to explore the effectiveness of different image features derived from anatomical MRI data. Scalar momentum, and other feature sets that made use of the background class, were generally found to be effective, irrespective of the target of prediction. There is a tendency for researchers to focus only on the tissues that constitute the brain (GM and WM), and to forget the additional class that accounts for the remaining “negative space”. Those analyses that combined kernel matrices derived from GM, WM and background (BG) – irrespective of whether modulation was used or not – tended to give more accurate predictions. Performance from scalar momentum was not highly dependent on the degree of smoothing, although the most accurate predictions were achieved using a smoothness of around 12 mm.

One result, which may surprise many, was that the widely used approach of using Jacobian-scaled (modulated) grey matter as features for pattern recognition was found not to be particularly effective. In all eight comparisons made in this paper, scalar momentum outperformed Jacobian-scaled grey matter. Similarly, it always outperformed the combination of Jacobian-scaled GM and WM. The probability of this happening by chance is the same as that of getting heads every time from eight coin tosses (p = 0.004). If one considers the five types of target variable (autism, schizophrenia, age, gender and BMI) to be a representative sample of the types of pattern recognition study usually performed using T1-weighted MRI, then there is some evidence that they would generalize to other related types of target variable (p = 0.03).

In this work, we were especially interested in more subtle anatomical variations that may be distributed throughout the brain, and are not easily visible by eye. There are likely to be areas of research that require a completely different approach from the linear methods used here. For example, studies on patients with stroke, or other pathologies that can appear in many different locations, would benefit from a machine learning approach that can handle nonlinearities. In other situations, a method such as Naive Bayes could even turn out to be most appropriate. Deep learning approaches may be most effective when numbers of subjects are extremely large. This work has only looked at linear Gaussian process methods, as well as support-vector machines for the classification tasks. It is possible that pattern recognition based on other approaches, such as sparsity inducing LASSO or Elastic-Nets, may benefit from very different feature representations. For example, machine learning using regularization based on an L_2_ smoothness penalty or on total variation regularization, is unlikely to benefit from having spatially smoothed feature maps. In general, the type of method used should be tailored according to available prior knowledge about how brains vary. We cannot truly say whether the findings from this work will generalize to a wide variety of other situations, but it is more parsimonious to assume a null hypothesis of no interaction effect (between the effectiveness of the features and the type of pattern recognition method used, or the task at hand) until evidence is provided that suggests otherwise.

The three datasets used in this work had been acquired on several different scanners, so it would seem unlikely that findings do not generalize over scanners. Image quality varied from dataset to dataset, and many of the scans in ABIDE had large regions of missing information (e.g. the cerebellum may not be included) and a variety of different image resolutions and artifacts. This suggests that the scalar momentum approach may also be effective for those mining hospital data.

Further work might be conducted to examine the scope of the findings and their dependence on software and settings. This work only assessed the behavior from tissue classes generated by SPM12, with settings all that their default value. Similarly, only the SPM12 geodesic shooting method for diffeomorphic image registration, with a particular set of default settings, was assessed. This approach is based around the LDDMM (large deformation diffeomorphic metric mapping) framework, where the concept of scalar momentum makes mathematical sense. Some investigators may wish to use features other than scalar momentum, in which case they could consider using warped data that has not been Jacobian-scaled, as this work found its performance to be similar to that of scalar momentum (i.e. there is not enough evidence to say whether it is better or worse). Alternatively, if Jacobian-scaled data is to be used, it may be advisable to also consider background as an additional tissue class. In every case for the Jacobian-scaled data, better performance was achieved using GM + WM + BG, than either GM alone, WM alone or GM + WM. Again, there is not enough evidence to indicate whether scalar momentum is generally preferable to kernel matrices constructed from Jacobian-scaled GM + WM + BG.

Pattern recognition techniques hold the promise of contributing to neuroscience, and could potentially be used in clinical practice. Before applying such approaches to large or valuable datasets, it is worth figuring out a few suggestions for best practice. Although machine learning practitioners often focus on the choice of machine learning algorithms themselves, in practice, feature engineering is really the key to their success (Domingos, 2012). Some readers may have their own hypotheses about feature representations that could be more effective than scalar momentum, in which case we urge them to test these hypotheses formally, and not simply accept that what everybody does is necessarily the most effective approach.

## Figures and Tables

**Fig. 14:**
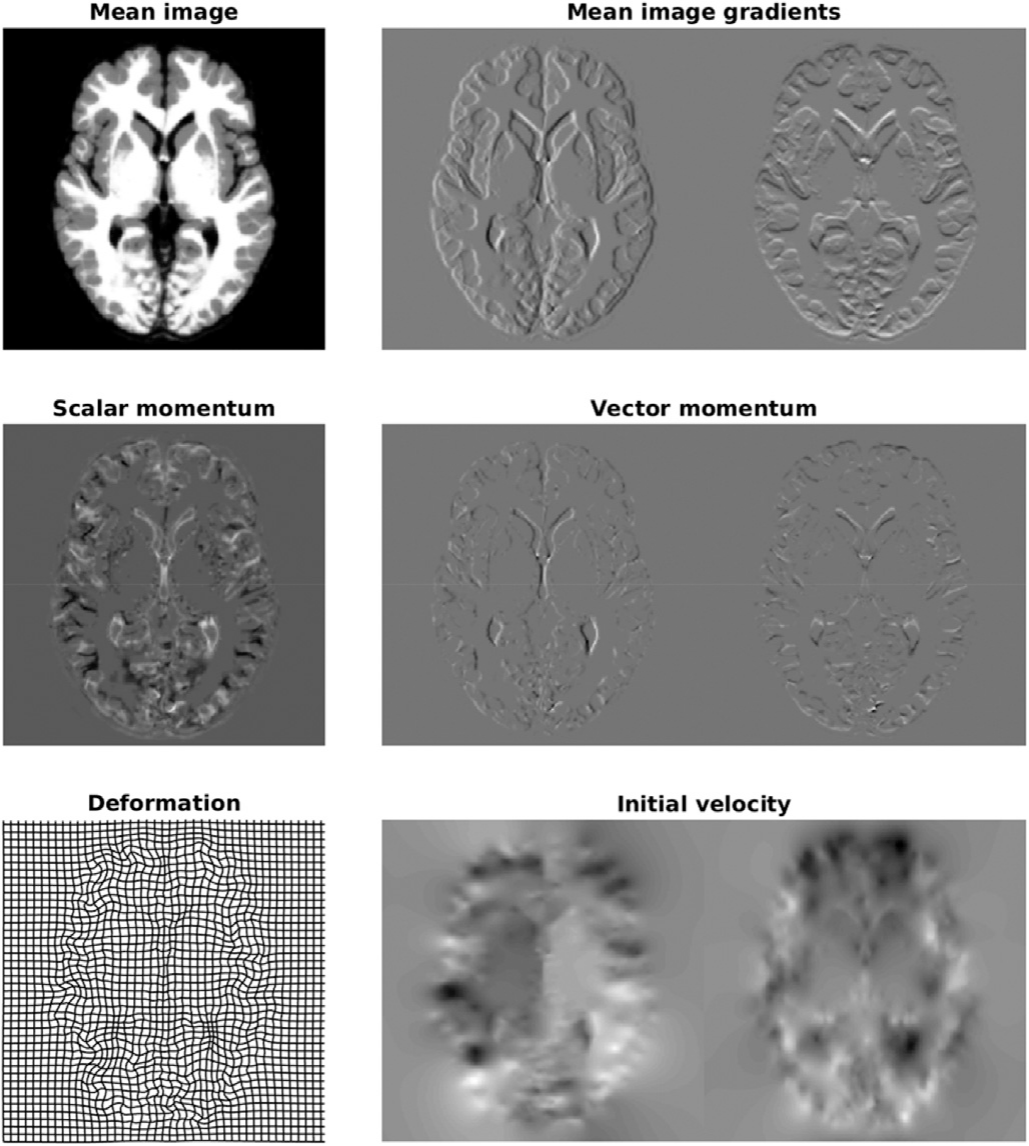
Illustration of how scalar momentum encodes diffeomorphic deformations. Top row: The template image (μ) and its gradients (∇μ ). Middle row: Scalar momentum ($a_{0}$) multiplied by the template gradients gives the vector momentum ($u_{0}=a_{0}\nabla \mu$). Bottom row: convolving the vector momentum with the Greens function of the differential operator used by the registration gives the initial velocities (v_0_ = (L^†^L)^g^ u_0_) from which both forward (φ) and backward ($\varphi^{-1}$) deformations may be computed.

**Fig. 15:**
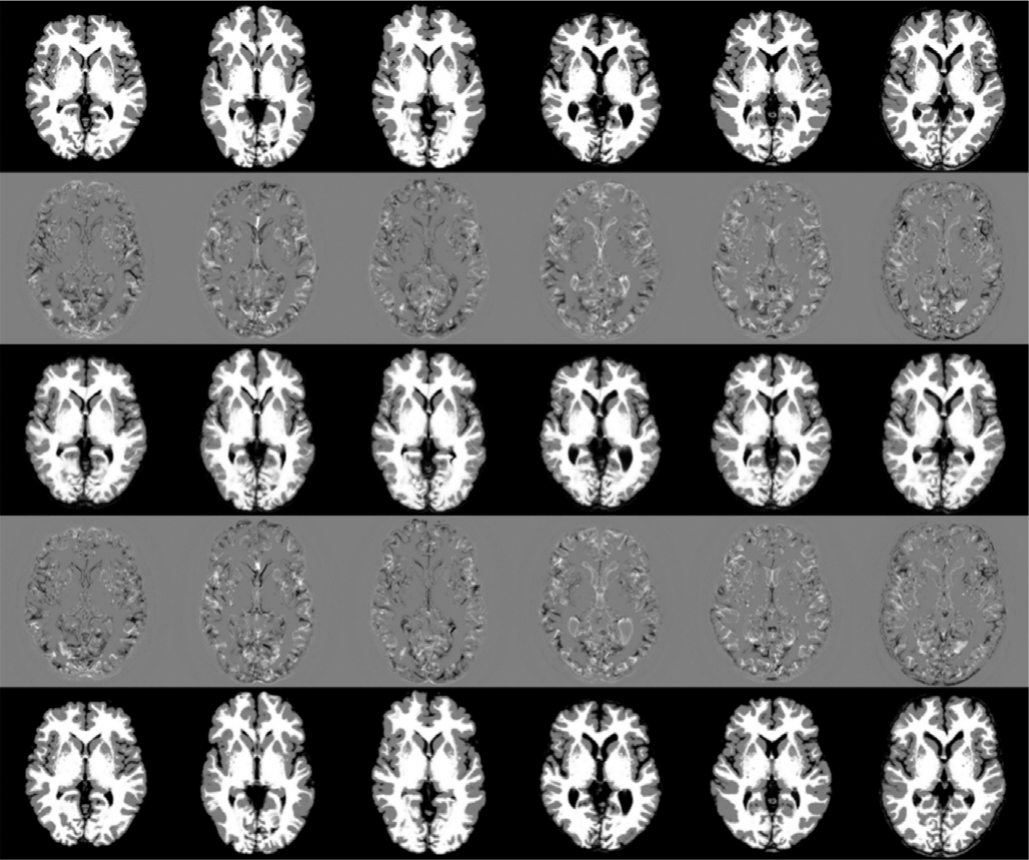
Illustration of how scalar momentum encodes the original data. First row: Five original 2D images. Second row: Scalar momenta obtained from aligning these to a common template. Because the scalar momenta (plus template) encode the diffeomorphic deformations (and the associated Jacobian determinants), it is possible to use these to warp the template to match the original data. Third row: Warped versions of the template show that alignment is reasonably good, but not an exact match. Fourth row: The deformations can also be used to warp the scalar momenta themselves, after they have been divided by the Jacobian determinants. Fifth row: Addition of images from the third and fourth rows shows that the original images are accurately encoded by the scalar momenta.

## Appendix

### (A) Scalar momenta

Inter-subject registration used the SPM12 Geodesic Shooting toolbox (Ashburner and Friston, 2011), which is a version of the Large Deformation Diffeomorphic Metric Mapping (LDDMM) algorithm (Beg et al. 2005), that has been reformulated to enable more efficient registration, both in terms of the number of iterations needed to converge and the amount of memory required.

LDDMM computes a diffeomorphic transformation *φ*: Ω→Ω between template image ***μ***, and a target image f (where spatial domain $\Omega \subseteq {ℜ}^{3}$), such that $f≅\mu \left(\varphi^{-1}\right)$. The curve defined between t∈[0,1], parameterizes the path by means of the ordinary differential equation $\dot{\phi_{t}}=v_{t}\left(\phi_{t}\right)$, where the diffeomorphism $\phi_{0}=Id$ (the identity) and $\varphi =\phi_{1}$ is defined as the end point of the path. The evolution is dependent on the velocity vector field of the flow of the deformation $v_{t}:\Omega \to {ℜ}^{3}$, and on t∈[0,1]. The diffeomorphism φ is obtained from

$$\varphi =\phi_{1}=\int_{0}^{1}v_{t}\left(\phi_{t}\right)dt$$

The objective is to estimate a series of velocity fields $\nu_{t}$ over *t*∈*[0,1]* by minimizing

$$\frac{1}{2}\int_{0}^{1}{||Lv_{t}||}^{2}dt+\;\frac{1}{2\sigma^{2}}{||f-\mu \left(\phi_{1}^{-1}\right)||}^{2}$$

Where L is a differential operator. The first term minimizes the squared distance of the deformation, which is the geodesic shortest path for the metric distance. The second term minimizes the difference between the warped template, $\mu \left(\varphi^{-1}\right)$, and the individual scan ***f***. The velocity at each time point may be derived from the initial velocity, so this initial velocity encodes the entire deformation via

$$v_{t}={\left(L^{†}L\right)}^{\text{g}}u_{t}\;;\;u_{t}=\left|D_{\phi_{\text{t}}^{-1}}\right|{\left(D_{\phi_{\text{t}}^{-1}}\right)}^{T}u_{0}\left(\phi_{\text{t}}^{-1}\right).$$

In the above notation, (*L*^†^*L*)^g^*u*_t_ denotes applying the generalized inverse of the operator (*L*^†^*L*) to the momentum field, *u*_t_, which in practice involves convolving with a Green's function. In this context, the key to the geodesic shooting approach lies in the fact that registration may be performed by only optimizing an initial velocity. Also, because the gradients are zero at the solution, we have

$$u_{0}=L^{†}Lv_{0}=\;\frac{1}{\sigma^{2}}\left|D_{\varphi }\right|\left(\mu -f\circ \varphi \right)\nabla \mu .$$

The entire set of images is aligned with the same template μ, so the vectorial part from the gradient of the template (∇μ) should be the same for each image of the sample. Therefore, the entire deformation may be computed from the template and a scalar momentum defined as

$$a_{0}=\frac{1}{\sigma^{2}}\left|D_{\varphi }\right|\left(\mu -f\circ \varphi \right).$$

Image registration involved a Gauss-Newton optimization of the initial velocity field, so accurately computing scalar momenta from Jacobian-scaled residuals requires the algorithm to have converged to within a fraction of a voxel from the optimum. These scalar momenta, along with the template, encode the deformation field, while simultaneously encoding the residual differences. As illustrated in Fig. 8, Fig. 9, this means they encode the information needed to reconstruct the original images (give or take some interpolation error) (Ashburner and Friston, 2011).

The above equations and illustrations in Figs. 14 and 15 assume that the image matching term of the registration involved minimizing an $L_{2\;}$ norm. The actual objective function used was based on simultaneously aligning several tissue classes, assuming they are drawn from multinomial distributions (Ashburner & Friston, 2009). Because of this, the “scalar” momenta are not actually scalar fields. The registration in the current study involved simultaneously matching grey matter (*c*_*1*_), white matter (*c*_*2*_) and an implicit background class (*c*_*3*_ = 1- *c*_*1*_ - *c*_*2*_), giving the following components for the scalar momentum:

$$\left|D_{\varphi }\right|\left(\mu_{1}-c_{1}\left(\varphi \right)\right)$$

$$\left|D_{\varphi }\right|\left(\mu_{2}-c_{2}\left(\varphi \right)\right)$$

$$\left|D_{\varphi }\right|\left(c_{1}\left(\varphi \right)+c_{2}\left(\varphi \right)-\mu_{1}-\mu_{2}\right)$$

Also note that in practice, a reduced form of the scalar momenta were used, so they had two, rather than three component fields. This involved working in the null space of the [1 1 1] vector, which saved memory and disk space, without changing any results.

Scalar momentum parameterizes the deformations, but it may also be conceptualized as the Jacobian-scaled residual error from the image registration. If not enough regularization is used, this error is likely to be dominated by noise in the image, which will also be reflected by noise in the resulting deformations. In all nonlinear image registration problems, there is a trade-off that needs to be sought between minimizing two terms: the term that measures the difference in appearance, and the term that measures the roughness of the warps. The registration is likely to be most accurate when these are optimally balanced, and hence the scalar momentum would also more accurately encode the relative shapes of the images.

## Footnotes

1 The concept of dependent and independent variables is tricky for purely observational data, where no direct interventions are made. The direction is straightforward for most task-based fMRI, as stimuli are usually presented in a way that is independent of BOLD signal. For many analyses relating to anatomical data (particularly where brain lesions are involved), perhaps image data should be considered independent variables (“causes”) and the symptoms treated as dependent variables (“effects”).

2 From http://dbm.neuro.uni-jena.de.

3 Available for download (without the requirement of including IXI on the author line of any publications) from http://biomedic.doc.ic.ac.uk/brain-development/index.php?n=Main.Datasets.

4 Dataset and details available from: fcon_1000.projects.nitrc.org/indi/retro/cobre.html.

5 Dataset and details available from: fcon_1000.projects.nitrc.org/indi/abide/.

6 SVM classification used Steve Gunn's MATLAB mex wrapper from http://www.isis.ecs.soton.ac.uk/isystems/kernel/, which calls Alex Smola's pr_loqo.c function to do the quadratic programming.
